# The Effects of eGovernment Efficiency on Subjective Wellbeing

**DOI:** 10.3389/fpsyg.2022.768540

**Published:** 2022-03-03

**Authors:** Mingyue Fan, Motswedi Epadile, Sikandar Ali Qalati, Naveed Akhtar Qureshi

**Affiliations:** ^1^School of Management, Zhenjiang, China; ^2^School of Finance and Economics, Zhenjiang, China; ^3^Department of Business Administration, Sukkur IBA University, Sukkur, Pakistan

**Keywords:** eGovernment efficiency, trust, utilization, subjective wellbeing, PLS-SEM (partial least squares structure equation model)

## Abstract

Undoubtedly, the internet has become the most convenient and efficient communication and service delivery channel adopted by most government agencies, referred to as eGovernment. This study explores how eGovernment efficiency influences users’ subjective wellbeing (SWB), using trust as a covert stimulus with the capacity to alter individuals’ overt behavior (utilization). Covert and overt stimuli act as significant factors influencing the relationship between citizens and the online environment, moderated by socio-demographic characteristics. Using situation–organism–behavior–consequence theory, we propose a research model consisting of online environment eGovernment efficiency (the situation) influencing trust development (the organism), which in turn influences utilization (the behavior), generating an impact on an individuals’ SWB (the consequence). We followed the structural equation modeling (SEM) approach to analyze the data survey *N* = 300, using Amos statistical techniques. Results reveal that the correlation between eGovernment efficiency and trust is positive and strong, trust and utilization is positive and moderate, and the correlation between utilization and SWB is positive and very strong. Stepwise regression analysis reveals that the control variables affect the relationship between eGovernment efficiency and trust. In the regression model: the highest education level explained 36% of the variance (model 1); adding age increased the variance explained to 39% (in model 2), and adding internet use frequency increased the variance explained to 41% (model 3). This study develops theoretical concepts of eGovernment use and how it affects citizens by indicating the psychological and behavioral situations as antecedents and mediators influencing SWB. It also provides practical suggestions for improving systems to correlate users’ feelings and behavior patterns to motivate trusting behavior, positively impacting users’ SWB to benefit citizens effectively.

## Introduction

Undoubtedly, the internet plays a crucial role in our lives. Whether used for work, business, education, communication, information, or relaxation, the internet is associated with technological innovation. This vast and complex environment motivated interest among many practitioners and researchers. The internet’s enormous potential has led to the appearance and promotion of several new concepts, such as electronic commerce for the private sector and government agencies (Carter et al., 2016). Due to social media popularity has also become common for government agencies to use social networks, such as Facebook, Twitter, and Instagram, to broadcast public updates and notices. Silcock (2001) defines electronic government (eGovernment) as an ecosystem that is a complex socio-technical system incorporating citizens, firms, and government agencies, which use electronic platforms to create and distribute value to its participants. Introduction of electronic services (e-services) made possible through inter-linked databases enabled easy storage and retrieval. This improved government agencies’ service delivery to benefit the government and citizens (Alshaher, 2021). Nowadays, eGovernment is considered a powerful, effective, efficient, and transparent tool that links government and non-government agencies and replaces offices’ time-consuming and expensive traditional infrastructure (Thompson et al., 2020). In a nutshell, it saves time, costs, and resources, ultimately improving governments’ efficiency (Joshi and Islam, 2018; Scholta et al., 2019). Initial expectations were that eGovernment would successfully replace these expensive and time-consuming traditional channels (van Dijk et al., 2007). However, Al-Hujran et al. (2015) argued that eGovernment is still experiencing implementation and adoption challenges. eGovernment projects’ failure is still a reality, from partial failures to complete rejections, negatively affecting end-user satisfaction due to not addressing real business needs. It is usual for a project to have positive and negative effects, but if the adverse effects dominate the positive ones, this represents a concern (Anthopoulos et al., 2016). According to Choi and Chandler (2020), the diverse behavioral and structural elements, either pulling or pushing forces of innovation, cause a spiral toward negative rather than positive, eGovernment dynamics. For example, traditional service objectives set out in the initial framework may not entirely be applicable in a web-enabled environment (Kaisara and Pather, 2011), which is the root cause of utilization problems (Li and Shang, 2020).

However, despite the availability of innovative technologies, government agencies face socio-economic, organizational, technological, and political challenges and barriers. These need to be addressed when developing, adopting, and implementing eGovernment systems and services (Carter et al., 2016). eGovernment involves massive investment in technological, financial, and infrastructural terms and necessitates significant shifts in public services’ institutional, behavioral, and legal aspects. These pose pretty complex and daunting challenges in developing countries. Further, there are no natural quick fixes to such challenges; in this context, Siddiquee (2016) argued that eGovernment development and change, especially in public administration, is a slow and incremental process. Moreover, eGovernment must be seen as a long-term initiative, requiring constant drive and significant investment, both in financial and non-financial terms. Globally, eGovernment achievements and challenges differ based on the country’s level of development (Alshehri and Drew, 2010). For instance, some developed countries, such as the United States, achieved high levels of eGovernment, whereas, in some developing countries, eGovernment achievements are slow due to economic and government instability (Li and Shang, 2020). According to Lessa (2019), extant literature shows that most developing countries experience slow growth regarding eGovernment initiatives, with some efforts ending in partial failure. Although eGovernment utilization and adoption challenges exist, some countries are making eGovernment services mandatory as a strategy to make citizens use eGovernment services (Ebbers and van de Wijngaert, 2020; Alkraiji, 2021). In Botswana, for instance, the Companies and Intellectual Property Authority (CIPA) has made company registration online-only, with no exceptions, making offline registration impossible (Mosimanegape, 2019). Citizens use eGovernment to access important information and services at their convenience, such as healthcare (Anthopoulos et al., 2016), tax returns (Mustapha and Obid, 2015; Immordino and Russo, 2018), driver’s license applications (Chelliah et al., 2016), and registering births and deaths or requesting identification documents online (Scholta et al., 2019). The Government of Botswana created a strategic plan [Integrated Government (1Gov) 2011–2016] (Churu, 2012) to create universal access to services through the use of appropriate strategies and techniques for efficient and effective service delivery. The planned service initiatives encompass national identity cards (OMANGs), passports, vital statistics registration (birth, marriage, death, etc.), land and property registration, motor vehicle registration, national statistics, business registration and licensing, and government core-service processes (HR, finance, procurement, project management, and knowledge management). However, Samboma (2019) reported that challenges, such as language barriers, poor infrastructure, lack of local authorities’ financial autonomy, and lack of trust, limit eGovernment effectiveness and its relationship with citizens. There are three mobile networks in Botswana with two mainly used prepaid data bundles, social network, and limited-time data bundles. Social network bundles are affordable and give limited access to Facebook, WhatsApp, Instagram, and Twitter. In contrast, limited-time data bundles are expensive and provide unlimited data access ranging from 1 to 24 h, valid for 30 days (Albano, 2021). Due to affordability, citizens mostly use social network bundles to enhance their day-to-day communication. However, as of the end of 2020, the eGov development index was 0.54 (ranked 115 of 193), with a 0.37 e-participation index (ranked 137 of 193) (United Nations, 2020). Since 71% of Botswana’s population lives in urban centers, 29% lives in rural areas (Kemp, 2021). Using a closed-ended questionnaire, we obtained the study sample in Maun city, the fourth most populated city, and the country’s tourism capital.

Features such as a safe and reliable system that is simple and easy to use are required to improve online trust. Trust plays a pivotal role in users’ acceptance and utilization of eGovernment and, consequently, its overall success (Pérez-Morote et al., 2020). To the best of our knowledge, although the effect of eGovernment efficiency on trust (Schierz et al., 2010) and utilization (Mensah, 2020) is clear, there are limited existing studies supported by a theory to rationalize how this combined influence affects individuals’ subjective wellbeing (SWB). Furthermore, de Róiste (2013) argued that there is still a need for more eGovernment investigation. Although this oversight is recognized, studies in this area are still in demand. Consequently, this study has three objectives: to understand the measures of eGovernment efficiency; to scrutinize the relationship between eGovernment efficiency and citizens in terms of the trust, utilization, and how this ultimately impacts individuals’ SWB; and to determine the influence of socio-demographic user characteristics on trust.

Employing the situation–organism–behavior–consequence (SOBC) model (Davis and Luthans, 1980), this study extends existing knowledge by explaining how the external environment “eGovernment efficiency” (the situation) affects the internal state of individuals’ “trust” (the organism), which in turn influences “utilization” (the behavior), triggering an impact on their “subjective wellbeing” (SWB) (the consequence).

This study makes several contributions by examining the SOBC theory’s inter-relationships. First, the efficiency of online public service delivery can positively affect the internal state of individuals and their actions. By concentrating on citizens’ psychological and behavioral constraints, this study can aid in the development of practical and effective public systems that consider emotions and views rather than focusing only on technological solutions, such as the introduction of complex methodologies. Second, this study illustrates the effectiveness of the SOBC theory in explaining the impacts of technology efficiency on individuals’ SWB, which is fundamental to the overall health of an individual. Third, it supports the mediating role of utilization on the relationship between trust and SWB. Finally, this study shows how demographic factors, such as age, the highest level of education, and internet access frequency, regulate users’ trust development. Above all, this study provides an enhanced description and estimation of how and why technology efficiency can positively impact individuals’ SWB.

The remainder of this paper is structured as follows: section “Literature Review” outlines the study concepts and theoretical background based on a review of the literature. Section “Research Methodology” details the hypothesis development and conceptual framework. Section “Results” comprises the methodology, including data collection and analysis methods, while the results are presented and analyzed in section “Discussion and Implication.” Finally, Section 6 discusses the results, provides conclusions, and details the study’s contributions and limitations.

## Literature Review

### eGovernment

eGovernment began about three decades ago to improve service delivery by making it practical and efficient (Ebbers and van de Wijngaert, 2020). According to Moon (2002), eGovernment includes five interaction levels: information provision, communicating with citizens, online transactions, integration of government agencies, and citizen participation. The basic idea of eGovernment was to replace time-consuming and expensive traditional front-desk channels, such as queues in offices, extensive telephone use, and intensive form-filling (Goodwin, 2010), with quick, accurate, and consistent online procedures (Pieterson et al., 2007). These changes would achieve benefits such as cost-efficient due to the diminished need for transportation to offices and reduced stationery expenses (Venkatesh et al., 2012). Hence, government agencies globally have invested in implementing and enhancing eGovernment systems (Alshehri and Drew, 2010). Although some citizens use eGovernment, most still prefer to interact with the government through traditional means (telephone or front desk), especially in developing countries (Madsen et al., 2020). However, numerous government agencies make some services accessible only online to encourage users to utilize eGovernment. In developed countries, such as Denmark and The Netherlands in Europe, many countries have made one or more services accessible through e-services only (Ebbers and van de Wijngaert, 2020), and citizens seem to have accepted it well. However, regarding developing countries, the same cannot be said. Research reports that overall, despite government efforts, eGovernment still creates significant utilization and adoption challenges (Papadopoulou et al., 2010; Al-Hujran et al., 2015).

### eGovernment Efficiency

eGovernment efficiency refers to improved public services, information transparency, and effective public participation with public sector management online, creating significant cost-savings for both citizens and government operations (Hackney et al., 2007). eGovernment efficiency mainly focuses on efficient service delivery that satisfies most users’ needs (Roman and Miller, 2015). Similar to Moon (2002)’s five eGovernment interaction levels, Holzer and Kim (2006) constructed a model to measure eGovernment efficiency consisting of five categories: privacy and security; usability; content; services; and citizen and social engagement. In the context of eGovernment studies, many researchers have explored eGovernment based on these efficiency measures, for example, usability (Gao and Lee, 2017); privacy and security (Janita and Miranda, 2018); content (Mokone et al., 2018); services (Tan et al., 2013); and citizen participation (Lee and Porumbescu, 2019). In the present study, eGovernment efficiency is considered a non-human environment situation. A non-human environment refers to situations or conditions likely to cause psychological and behavioral effects among individuals (Searles, 1960).

### Subjective Wellbeing

According to Chen et al. (2013), psychological consequences significantly influence individuals’ wellbeing. SWB is essential to overall wellbeing because it comprises cognitive elements of life satisfaction (Diener et al., 1998). It plays a vital role in the positive development of an individual as an indicator, a moderator, a mediator, a predictor, or an outcome (Park, 2004). The theory of core affect by Russell (2003) states that various internal and external factors influence reflexes, perception, cognition, and behavior. Still, people do not have direct access to these causal connections. As a result, core affect can be experienced as a free-floating (mood) state or attributed to a specific cause and, thereby, an emotional episode. SWB is a psychology field that attempts to examine and understand how people evaluate their lives regarding positive and negative emotions (Peterson et al., 2005; Diener, 2009). Negative emotions are related to social, psychological, and behavior problems (Peterson et al., 2005), while positive emotions are related to good adaptation and optimum mental health (Proctor et al., 2010). SWB has three components: positive affect (PA); life satisfaction (LS); and negative affect (NA) (Andrews and Crandall, 1976). Individuals have positive SWB if they experience LS and frequent PA, such as happiness and optimism, and infrequent NA, such as rage, annoyance, and sadness. Feelings associated with PA and LS empower people to overcome complications positively and accomplish what they want in life (Diener and Chan, 2011). NA feelings negatively influence individuals’ wellbeing, creating extra pressure and anxiety both on an individual’s body and mind, which might lead to health issues if the strain becomes long-lasting or overwhelming (Xu and Roberts, 2010). Lawless and Lucas (2011) argued that, wherever there is a positive influence on life satisfaction, there is subsequently greater life expectancy, lower levels of homicides, as well as lower rates of liver and heart-related diseases. Governments control essential services citizens need to generate and secure their economic situations and meet their basic needs. In this context, Diener (2016) advocated that an individual’s levels of SWB are influenced both by internal factors, such as behavior and viewpoint, and external factors, such as the environment in which they live, and, most importantly, their ability to meet their basic needs. Therefore, we argue in this paper that eGovernment experience has an essential effect on citizens’ wellbeing because every situation that affects an individual affects their wellbeing (Diener et al., 1985).

### The Situation–Organism–Behavior–Consequence Model

**FIGURE 1:**
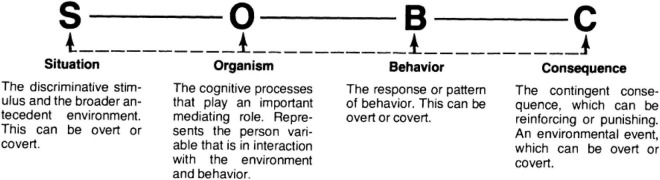
The SOBC model.

SOBC theory states that different elements of the environmental situation (S) affect the internal states of organisms (O), which influences specific behavioral responses (B), triggering consequences (C) as a result (Davis and Luthans, 1980). According to Whelan et al. (2020), the SOBC model is a more multifaceted mechanism of human behavior that modifies and extends the antecedent stimulus–organism–response (SOR) (Mehrabian and Russell, 1974) and antecedent–behavior–consequence (ABC) (Skinner, 1963) models. These authors further assert the importance of identifying the effects of covert stimuli, such as thoughts, expectations, and dreams, as the crucial mediating role for O and B between S and C. Similarly, recognizing utilization behavior is essential in adjusting the relationship between the environment and people. Figure 1 depicts the SOBC model (Davis and Luthans, 1980).

This study employed the SOBC model for several reasons. First, it is suitable for studying the influence of technology use on citizens’ emotions and behavior because it provides a robust theoretical approach for developing and validating an appropriate research model focusing on people and their environment (Whelan et al., 2020). Second, the SOBC model provides a structured procedure that specifically assists in examining how individuals’ internal state of mind mediates the relationship with their environment, which subsequently impacts their SWB. According to Van Hoorn (2007), SWB is a self-report technique for measuring wellbeing based on physical surroundings and social situations. Tuncer et al. (2005) also asserted that these situations could influence people’s behavior. Cooper et al. (2007) stated that behavior takes place in the form of a process. Therefore, firstly, environments affect people’s psychology, then they react, affecting their wellbeing. Research has highlighted trust as the primary mediator between technology (an online environment) and its utilization (Colesca, 2009). Similarly, the SOBC model uses the environment to determine how it affects people; thus, our study expects trust to mediate the relationship between eGovernment efficiency and utilization. eGovernment studies primarily implemented technology acceptance models (TAMs), such as the unified theory of acceptance and use of technology (UTAUT) (Venkatesh et al., 2011) and TAM (Lin et al., 2011) to explore and explain users’ intentions to utilize e-services and subsequent usage behavior. Thus, by using the SOBC model, this study extends and diversifies the theoretical and practical perspectives in the study of eGovernment dimensions.

### Hypothesis Development and Conceptual Framework

This section describes how this study selected and aligned variables with the SOBC theory. We combined prior research insights with the SOBC theory to develop this study’s research model. Malodia et al. (2021) conducted interviews with government officials, citizens (urban and rural), technology experts, senior policy-makers, government agencies, and academic researchers from November 2016 to January 2019. With the evolution of eGovernment literature from 2002 to 2019, the author reported that efficiency, trust, citizen satisfaction, infrastructure, culture context, citizen readiness, and citizen empowerment contribute to the successful implementation of eGovernment. These factors align with the five Moon (2002)’s eGovernment interaction levels and Holzer and Kim (2006)’s eGovernment efficiency factors.

**FIGURE 2:**
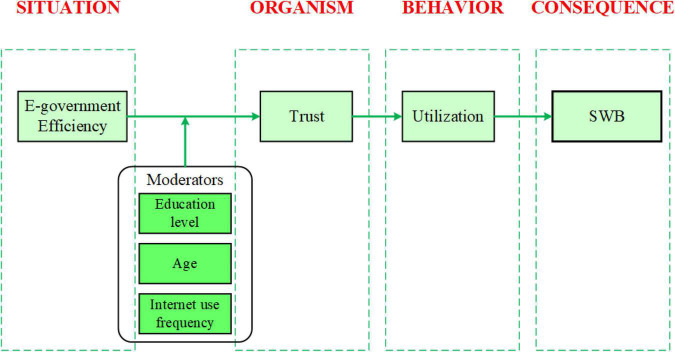
Research model.

Consequently, this indicates that eGovernment efficiency sums up almost all aspects contributing to eGovernment success. Therefore, this research considers efficiency a robust environment antecedent influencing the relationship between eGovernment and citizens. Similarly, prior research has heavily pointed to trust in different forms as major cognitive processes representing the person variable in regards to e-services utilization and adoption (Colesca, 2009; Schierz et al., 2010; Cheng et al., 2017a,b). Since our main objective is how this affects SWB, wellbeing is fundamental to health and happiness. Having a well-adapted and robust sense of wellbeing can help individuals overcome difficulties and help them achieve their goals in life (Diener and Chan, 2011), and reduce levels of homicides, liver, and heart-related diseases (Lawless and Lucas, 2011). Therefore, this study identified eGovernment efficiency, trust, and utilization as significant factors contributing to SWB. Figure 2 represents our research model, in which we propose the relationships between the covert stimuli of eGovernment efficiency, trust, utilization, and SWB. In our model, eGovernment efficiency represents the situation (S), trust represents the organism (O), utilization represents the behavior (B), and SWB represents the consequence (C). Users measured eGovernment efficiency based on their expectations regarding the five efficiency categories: privacy and security; usability; content; services; and citizen and social engagement (Holzer and Kim, 2006). Within the psychology field, an expectation is a strong belief that something will occur or be the case (Driskell and Mullen, 1990; Martin et al., 2001). Citizens’ expectations and level of satisfaction influence eGovernment use; when these are high, their trust and intention to use eGovernment services also increase (Khan et al., 2020; Rosenberg, 2020). Finally, SWB, this study’s dependent variable, refers to the psychology field that attempts to examine and understand how people evaluate their lives based on life satisfaction, joy, and thinking and feeling that life is going in the right direction (Diener, 2009).

#### eGovernment Efficiency and Trust

As trust is a complex and abstract concept, it also becomes complex to define and identify the elements that construct it (Wang and Emurian, 2005). Milloy et al. (2002) state trust in transit, such as trust in the infrastructure, and trust in usage and access, such as trust in the organization and how it handles information. Warkentin et al. (2002) defined technology trust in an organization and trust in government as vital concerning electronic services because they are important determinants of service performance. Although online trust shares similar features to offline trust, some essential differences are unique in an online environment. These features include a robust, safe, and reliable system that makes them simple and easy to use are requirements to improve trustworthiness (Singh and Sinha, 2020); this is in line with Holzer and Kim (2006)’s five eGovernment efficiency measures. For instance, Colesca (2009) stated that before users trust eGovernment initiatives, they must believe that the government possesses the technical and managerial resources required to implement and secure these systems, meaning that citizens trust eGovernment based on its efficiency in terms of security and privacy. Additionally, Schierz et al. (2010) validated a hypothesis on the significant positive influence of technology efficiency on individuals’ cognition, such as perceived trust. They further defined perceived trust as an emotional state that encourages one party to develop trust based on the other party’s behavior. Based on this theory, the present study users’ trust development is a cognitive process representing the internal state of an individual (O), which is influenced by eGovernment efficiency (S) (Reichheld and Schefter, 2000). Therefore, we theorize that:

H1. eGovernment efficiency positively influences trust development.

#### Trust and Utilization

Consistently, trust is the leading influencer of e-service usage and adoption (Carter et al., 2016). Previous studies drawing on eGovernment theory have reported the linking role of users’ trust on eGovernment as having a positive effect on adoption (Bélanger and Carter, 2008; Alzahrani et al., 2017). These results indicate that the missing link in eGovernment adoption lies within the antecedents of trust, security, and privacy, which are considered the main leading factors to eGovernment’s successful use and adoption. Citizens’ intentions to utilize e-services and subsequent satisfaction levels influence eGovernment adoption (Janita and Miranda, 2018). Utilization is the behavior of making practical and effective use of something (Julnes and Holzer, 2001), leading to adoption (Alzahrani et al., 2017). According to Califf et al. (2020), trust is the confidence level that a person will find what is expected rather than what is feared, representing a behavior influencing one’s intention toward utilization. Bozic and Kuppelwieser (2019) described trust as a state of mind-controlling the intention to accept vulnerability based on positive opportunities associated with utilizing anything (Singh and Sinha, 2020). Mofleh and Wanous (2008) performed a multiple linear regression analysis to investigate the link between intention to use eGovernment and trust. Their findings correspond with those of Meftah et al. (2015), indicating that trust in the internet and trust of the government agency has a significant effect in predicting citizens’ intention to utilize eGovernment services. According to Kumar et al. (2017), trust positively impacts citizens’ attitudes and behavior concerning eGovernment utilization. Since trust is a covert state of mind (O), causing a person to develop utilization habits (B), this study proposes the following hypothesis:

H2. Trust development positively influences utilization behavior.

#### Utilization and Subjective Wellbeing

System utilization mainly depends on Holzer and Kim (2006)’s five efficiency measures. Suppose the system has good content, is easy to interact with, is secure, ensures users’ privacy, and allows communication between government representatives and users. In that case, users are highly likely to utilize it. The government deals with essential services citizens need to generate and secure their economic situations to meet their basic needs. Therefore, if such services are accessible online, the service-delivery satisfaction level also influences their happiness and positively affects individuals’ SWB. Various studies have indicated that the environment plays a significant role in individuals’ SWB (Diener et al., 2003). SWB refers to individual emotional responses, domain satisfactions, and global life satisfaction judgments (Diener et al., 1985). According to Kumar et al. (2017), satisfaction is the positive result of comparing the actual service performance to the expected performance. Satisfaction occurs when service performance meets user expectations (Janita and Miranda, 2018). eGovernment utilization benefits include time-savings, cost-savings, and effective service delivery, which improves citizens’ lives (Venkatesh et al., 2012). Suppose users find it hard to use an organization’s poorly designed website without accommodating or considering different services for different users. In that case, some users are likely to be annoyed, frustrated, and disappointed (Allahawiah, 2013).

On the other hand, if it is easy to use, well designed, safe, and caters to different users’ needs, almost all users will be happy and satisfied. However, Kahneman and Krueger (2006) suspect many policymakers to be more comfortable minimizing specific concepts of hardship instead of boosting a nebulous idea of happiness and citizen satisfaction. Therefore, individuals’ experience interacting with eGovernment information systems may hugely influence citizens’ wellbeing because every situation affecting an individual affects their wellbeing (Diener et al., 1985). Therefore, continuous utilization occurs due to satisfaction, leading to adoption, which positively affects users’ SWB. Accordingly, we theorize that:

H3. Utilization behavior positively influences subjective wellbeing (SWB).

#### The Moderating Role of Socio-Demographics

Citizens’ demographic characteristics, such as age, gender, highest education level, and internet access frequency, predict online trust. Therefore, there is a need to comprehensively identify and understand their effects on trust to establish and maintain a better relationship between users and eGovernment. Since the internet is intensively used in schools, especially in tertiary institutions, for research and other academic purposes, this exposure to the online environment leads to the highest level of education impacting online trust. Following Wei et al. (2010), this theory applies to information and communication technology (ICT) usage, indicating that environmental factors, such as schools and companies providing frequent ICT access and training, affect ICT self-efficacy, which affects people’s lives covertly and overt behavior. Hong (2013) argued that internet access frequency, mainly influenced by social media use, positively influences trust in eGovernment, primarily because citizens who interact with the government through social media are most likely to trust other eGovernment platforms than those who do not. The author also argued that in all eGovernment platforms, social media might backfire if citizens’ expectations are insufficiently met.

Statistically, age is proven to significantly affect the decision to trust and adopt eGovernment (Welch et al., 2005). Young users are more likely to trust eGovernment services compared to older users because young people tend to be more open to the idea of using internet services overall than older people (Colesca, 2009). The younger generation intensively uses smart electronic devices connected to the internet in their daily activities to stay connected to friends and family *via* social media platforms (Whelan et al., 2020) and surf the internet, given its perceived usefulness for social aspects such as fashion. Such experiences play a vital role in influencing the strength of the relationship between technology acceptance and trust (Yang and Shih, 2020). In this way, the younger generation might expect eGovernment systems to behave similarly to what they already know regarding features and usability (Olson et al., 2011); when they are similar, their expectations are likely to be met and highly satisfied.

Precisely, prior literature shows that demographics, such as gender and age (Lin et al., 2011), media use and social perception (Grimmelikhuijsen et al., 2013), and education (Choudrie and Dwivedi, 2005), influence the strength of the relationship between people and their environment. Thus, we theorize that:

H4. Age, gender, internet access frequency, and highest education level moderate the relationship between eGovernment efficiency and trust.

## Research Methodology

We adopted an online survey as a convenient data collection method. It overcomes the distance barrier (Wright, 2005), granting simultaneous access to a large population (Fan et al., 2021; Ostic et al., 2021). This study shared a closed-ended five-point Likert-type scale ranging from (1 = strongly disagree to 5 = strongly agree) *via* email and social media to collect data. We adopted all study measures from prior literature, eGovernment efficiency (Elling et al., 2012, 2007), trust (Li et al., 2008; Lean et al., 2009), utilization (Lean et al., 2009; Shareef et al., 2011), and SWB (Diener et al., 1985; Neto, 2011). Following a pilot study of 80 respondents to verify reliability (Cronbach’s alpha ≥ 0.70) and validity [Pearson’s correlation, *p* < 0.01 (2-tailed)], the questionnaire was considered reliable and valid (Chelliah et al., 2016). Due to time and often internet-/network-related challenges experienced in the country (Samboma, 2019), data collection was conducted from July to November 2020, yielding a sample of 300 individuals from Maun city. The five-month timeframe was also to cater to citizens with limited internet access. Since there internet access difference, mainly due to occupation, purposive and cluster sampling was implemented to group citizens into four sampling units (university students, government employees, private-sector employees, and unemployed) to logically represent the population in an attempt to mitigate common method bias (CMB) (Jordan and Troth, 2020). Suggested by when using structural equation modeling (SEM) to detect an effect size, Cohen’s *d* = 0.40 with 80% power (alpha = 0.05, two-tailed), g*power linear multiple regression sensitivity power analysis suggests we would need a minimum total sample size of 96 participants. Published SEM research typically uses 200–400 cases to fit models (Hox and Bechger, 1998). Because our study involves latent variables and a heterogeneous population, we increased our sample size to establish valid results (Hair et al., 2019). We used IBM SPSS Statistics 23 and Amos 23.0 software as statistical data analysis tools to measure the effect of eGovernment efficiency on SWB for the following statistical analysis methods:

1. *Descriptive statistical analysis:* provides synopses concerning the sample and measures. Frequency analysis was implemented to analyze demographic and research variables to help analyze the results and draw conclusions.
2. *Structural equation modeling (SEM):* a statistical analysis method popular in social science research that integrates path analysis, factor analysis, and multiple regression analysis. In this study, the SEM methods included factor analysis to examine the relationship between the observed variables and the latent variables and multiple regression analysis to determine whether demographic variables predict the trust variable.
3. *Factor analysis:* a statistical analysis that reduces variables into a smaller number of factors. Before performing factor analysis, the Kaiser–Meyer–Olkin (KMO) test and Bartlett’s test of sphericity should be performed to determine whether the data are suitable for factor analysis.
4. *Stepwise multiple regression:* a statistical method that uses an automatic procedure to fit regression models in which the choice of predictive variables is entered into the regression equation once. Hagen (2015) stated that, in regression analysis, three results are most important: the multiple correlation coefficient (*R*), the coefficient of determination (*R*^2^), and the analysis of variance (ANOVA).

## Results

### Demographic Descriptive Statistics

Out of 500 questionnaires shared, a total of 300 respondents completed the survey from the share links and used for the analysis, a response rate was 60%. As the questionnaire fields were validated and there were no missing data, all responses received were usable. Approximately 73% of respondents had tertiary-level education, 19% high-school, 6% junior-school, 2% primary-/elementary-school, and none fell under the never attended school category. Regarding employment, approximately 18% of respondents were government workers, 26% were private-sector employees, 31% were university students, and 25% were unemployed. About 43% of the respondents were female, and 57% were male. The minimum respondents’ age was 25–34 years, and the maximum was 45–54 years. Almost 78% of respondents accessed the internet daily, 19% twice a week, 1% fortnightly, and 2% once a month. Supplementary Appendix 1 shows the respondents’ demographic attributes.

### Descriptive Statistics of the Scale Variables

This study’s descriptive statistics results for the 18 items for the four constructs are shown in Supplementary Appendix 2, encompassing the minimum value, range value, maximum value, mean value, standard deviation, skewness, and kurtosis. According to George (2011), when proving a normal univariate distribution, values for asymmetry and kurtosis between –2 and + 2 are considered acceptable. Similarly, Bryman (2012) argued that data the skewness values between –2 and + 2, and kurtosis values between –7 and + 7 are considered normal. This study skewness and kurtosis values follow the normal distribution, and the results are detailed below.

Respondents believe that eGovernment efficiency is characterized by websites or systems that are easy to use, with a clear homepage, sufficient and relevant information, as well as a search option to aid in quick access to the desired output. According to Immordino and Russo (2018), users assess e-services using quality aspects such as the service quality, system quality, and information quality of any e-service; these aspects are crucial concerning e-services’ success. Similarly, prior studies highlighted up-to-date information, usefulness, ease of use, web interface, and search engine as attributes that users consider in assessing competence (El-Kassem et al., 2020; Thu et al., 2020).

Regarding trust, the findings reveal that respondents have confidence in government agencies that take full responsibility for any type of security breach during interactions/transactions on the website and do their best to help users if they have a query. Systems or websites are considered more reliable and convenient than physical offices. Prior research has also indicated that reliability, validity, security, and privacy are essential antecedents of eGovernment trust (Thu et al., 2020; Zhu et al., 2020). Users’ covert or overt attributes, such as beliefs and attitudes related to agencies having the best interests of society and its constituents at heart, significantly impact trust and intention to use e-services (Alryalat et al., 2020; Pérez-Morote et al., 2020).

About utilization, respondents believe that interacting with government websites to receive government services enhances an individual’s or an organization’s social status. In terms of the skills needed to interact with online systems, respondents acquire them from computer technology at their workplace, institution, or home. Respondents also believe that websites provide a wider choice of interactions with different functions than interactions within a physical government office. Prior research has shown that personal outcome expectations act as cognitive abilities influencing users to utilize eGovernment services (Alruwaie et al., 2020). Additionally, Mensah et al. (2020) argued that obtaining the desired benefits positively influences intentions to use eGovernment services.

Finally, concerning SWB, respondents believe that, in most ways, online government services are close to ideal and only if their experience with online governance is excellent. Therefore, online service delivery offers essential services needed in daily lives, and only a few critical things need to be changed in existing platforms to perfect the efficiency. Similarly, prior studies have shown that eudaimonia and life satisfaction are cognitive evaluations of SWB, while happiness and anxiety are effective evaluations (Mouratidis, 2020, 2019). Happiness, anxiety, and satisfaction fall within the three components of SWB evaluation (PA, LS, and NA). Therefore, integrating both cognitive and affective measures of SWB to satisfaction with life domains yields essential insights.

### Reliability and Validity Analysis

Reliability is a method of assessing the consistency of a measure. This study evaluated the multiple Likert-scale items’ internal consistency using Cronbach’s α coefficient. The accepted Cronbach’s α value is 0.70 and above (Peters, 2014). The reliability analysis results are shown in Table 1. The Cronbach’s α value for each type of factor and the total sample is above 0.7, signifying a strong consistency among the items.

**TABLE 1:** Results of the reliability analysis.

Element	Cronbach’s α	No. of items
eGovernment efficiency	0.709	5
Trust	0.849	4
Utilization	0.702	4
Subjective well-being (SWB)	0.766	5
Total sample	0.879	18

In this study, Bartlett’s test of sphericity was used for the validity analysis, which evaluates whether the data matrix is an identity matrix. The KMO test was used to measure the sample adequacy for each variable in the model and the complete model. If Bartlett’s test of sphericity’s value is less than 0.5, this means that the data has good construct validity. Similarly, if the KMO value is at least 0.50, the sample is adequate, with KMO values closer to 1 representing greater adequacy (Antony and Visweswara Rao, 2007). The results are shown in Table 2. The KMO statistics values are greater than 0.7. The F value of Bartlett’s spherical test equals 0.000, which means that the sample data is adequate and has good construct validity, hence being suitable for factor analysis. Following Jordan and Troth (2020), this study evaluated CMB using Harman’s single factor in SPSS Statistics 23 (33%) and the common latent variable test in Amos 23.00 (30%). The results are shown in Table 3. The tests variance is less than 50% in both tests, suggesting that CMB does not affect this study (Meade et al., 2007).

**TABLE 2:** Results of the validity analysis.

Element	KMO	Bartlett’s test	*p*-value
eGovernment efficiency	0.756	235.267	0.000
Trust	0.821	494.497	0.000
Utilization	0.736	217.722	0.000
Subjective well-being	0.788	370.189	0.000

**TABLE 3:** Results of common method variance test.

Test	Percentage of variance
Harman’s single factor	33
Common latent variable	30

### Factor Analysis

Factor analysis aims to reduce the number of measurement variables and reduce multicollinearity among the main variables for further analysis through model regressions. Principal component analysis was used to determine the factor loadings using direct oblimin rotation, with the criteria that the extraction eigenvalue should be greater than 1 and the factorial loadings should be 0.5 or above (Hair et al., 1998). The factor analysis results are shown in Table 4. One item from eGovernment efficiency (Q5) failed to meet the minimum cutoff criteria and was dropped, reducing the number of variables from 18 to 17.

**TABLE 4:** Results of factor analysis.

Factor	Item
eGovernment efficiency	Q1, Q2, Q3, Q4
Trust	Q6, Q7, Q8, Q9
Utilization	Q10, Q11, Q12, Q13
Subjective well-being (SWB)	Q14, Q15, Q16, Q17, Q18

### Structural Equation Modeling

**FIGURE 3:**
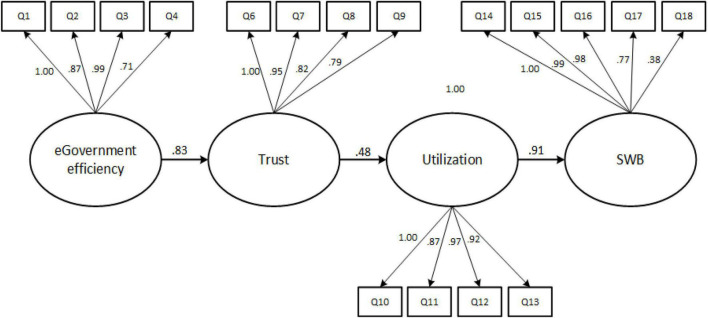
SEM effect of eGovernment efficiency on SWB.

SEM was conducted using Amos 23 software based on the factor analysis results. The maximum likelihood estimation method was utilized for the confirmatory factor analysis in a linear equation with the dependent variable Y having three dependent variables. The structural equations causal model is shown in Figure 3. This study established the following model fit criteria: χ^2^/df < 3; RMSEA < 3; AGFI, GFI, NFI, IFI, CFI, and RFI > 0.90; and PNFI > 0.05 (Little, 2014). The thresholds of the model fit are as follows: χ^2^/df = 1.67; RMSEA = 0.047; AGFI = 0.911; GFI = 0.935; NFI = 0.900; IFI = 0.952; CFI = 0.979; RNF = 0.935; and PNFI = 0.720. Thus, the model fit criteria are met.

The parameter estimation results (Supplementary Appendix 3) show correlations between the variables. Correlations that are ≤ 0.35 are considered weak correlations, 0.36–0.67 moderate correlations, and 0.68–1.0 strong correlations, with r coefficients > 0.90 very strong correlations (Taylor, 1990). The results are as follows.

The correlation between eGovernment efficiency and trust is positive and strong, supporting H1: *r*(298) = 0.83, *p* < 0.001. Respondents develop confidence in public online services depending on their effectiveness and efficiency. Easy to use systems, with a simple interface and without information overload on the home page, make a quick orientation possible. Systems with sufficient information and an effective search engine assist in quick access to the right information. Such systems make respondents feel confident in relying on government agencies to do their best to help users feel safe and secure when accessing services online. Similarly, using TAM, Pérez-Morote et al. (2020) conducted a multiple regression analysis where the variable “trust in government” obtained a significant beta coefficient (β = 0.314, *p* < 0.00), indicating a positive relationship between trust in government and citizens’ use of eGovernment. Using the mobile-government adoption model (MGAM), a model integrating the UTAUT model and the e-government adoption model (GAM) (Almaiah et al., 2020), conducted SEM path analyses, showing that perceived service quality has a significant effect on trust in mobile-government. Their results indicated a significant positive relationship between perceived service quality and increased perceived trust among users (β = 0.501, *p* < 0.001).

The correlation between trust and utilization is positive and moderate, supporting H2: *r*(298) = 0.48, *p* < 0.001. When respondents feel that the online systems are more reliable than physical government offices and that government agencies take full responsibility for any security breach, it motivates them to use them effectively. These results are in line with those of Zhu et al. (2020) in that trust in technology is positively related to citizens’ intention to use e-services (β = 0.473, *p* < 0.000). Correspondingly, Mensah et al. (2020) stated that government trust is positively related to the behavioral intention to use. The author utilized a structural model extended from the unified model of electronic government adoption (UMEGA), a parsimonious model based on the eGovernment-specific appropriate context with constructs originating from technology adoption models (Dwivedi et al., 2017), eGovernment services, with the results being positive and significant (β = 0.387, *p* < 0.05).

The correlation between utilization and SWB is positive and very strong, supporting H3: *r*(298) = 0.91, *p* < 0.001. Respondents access public services through government websites to enhance their social status, either individually or for their organization. It provides a wider choice of interactions with different functions from the comfort of their offices/homes compared to interactions within the physical government office. In their integrated model, Pérez-Morote et al. (2020) used expectancy disconfirmation theory (EDT) to prove that technology use (eGovernment and social media) expectations affect satisfaction end-result. Whelan et al. (2020) also applied the SOBC paradigm to depict how social media use as covert behavior influences academic performance as a consequence. Similarly, this paper shows that overt or covert behavior regarding technology use influences SWB.

### Stepwise Regression of the Moderating Role of Age and the Highest Level of Education

A multiple linear regression done by Khamis et al. (2003) revealed that the higher the R2 value, the better the model fit. Similarly, Hagen (2015) stated that, in regression analysis, three results are most important. The first is the multiple correlation coefficient (R), which measures the correlation strength between the predicted and observed variable; the higher the R-value, the stronger the correlation (Cook, 1977). The second is the coefficient of determination (R2), which explains to what extent independent variables can explain variation among dependent variables (Menard, 2002). The third is ANOVA, which is the analysis of variance and which tests two essential aspects. First, ANOVA determines whether the regression model predicts an outcome variable or not using degrees-of-freedom and F statistics. Second, it confirms whether the results are statistically significant and whether the results can be generalized to the country’s entire population.

This study adopts a Stepwise multiple regression approach to evaluate whether sector, internet use frequency, the highest level of education, gender, and age socio-demographic variables predict trust. The results are shown in Table 5. In model 1, the highest education level was entered into the regression model, and this significantly predicted trust: β = 0.603, *t*(298) = 13.06, p < 0.001. Highest education level also explained a significant proportion of variance in trust: R2 = 0.36, *F*(1, 298) = 170.51, *p* < 0.001. In model 2, age was entered into the regression model, and this significantly predicted trust: β = –0.198, *t*(297) = –4.02, *p* < 0.001. Age increased a significant proportion of variance in trust: R2 = 0.39, *F*(2, 297) = 97.66, *p* < 0.001. In model 3, internet use frequency was entered into the regression model, and this significantly predicted trust, β = 0.119, *t*(296) = 2.18, *p* < 0.001. Internet use frequency also increased a significant proportion of variance in trust: R2 = 0.41, *F*(3, 296) = 67.51, *p* < 0.001. Sector and gender were not entered into the regression model [sector (*t* = 0.41, *p* > 0.05); gender (*t* = 1.22, *p* > 0.05] as they are non-significant in trust prediction (Fritz and Berger, 2015). The best regression model for predicting trust is model 3, with the highest education level, age, and internet use frequency as predictors; it is the most significant and has the highest R2 value (Li and Pham, 2019). Similarly, integrating five theories being; the digital divide theory, TAM, diffusion of innovation theory, and expectancy disconfirmation theory, Pontones-Rosa et al. (2021) conducted a factor analysis using the principal components method with Varimax rotation and bivariate analysis with contingency tables between the satisfaction variables and the municipal, demographic, socio-economic, and technological variables the ICT access, quality, and use. Their results had high factor loadings or significant weights, highlighting different population profiles according to satisfaction levels with ICT development and confirming their hypothesis.

**TABLE 5:** Summary of stepwise regression analysis for variables predicting trust (*N* = 300).

	Model 1			Model 2			Model 3		
Variable	B	SE B	B	B	SE B	B	B	SE B	β
Highest education level	0.47	0.04	0.60**	0.41	0.04	0.52**	0.36	0.04	0.46**
Age				–0.13	0.03	–0.20**	–0.13	0.03	–0.19**
Internet use frequency							0.11	0.05	0.12*
*R* ^2^		0.36			0.40			0.41	
*F* for change in *R*^2^		170.51**			97.66**			67.51**	

*p < 0.05, **p < 0.01.

Model 3 shows that the highest education level coefficient is positive and significant (*B* = 0.461, *p* < 0.001), indicating that trust also increases as the education level increases. The age coefficient is negative but significant (*B* = –0.187, *p* < 0.001), meaning that as age increases, trust decreases. Finally, the internet use frequency coefficient is positive and significant (*B* = 0.119, *p* < 0.001), which means that as internet use frequency increases, so does trust. Courville and Thompson (2001) suggested that, in multiple regression analysis, the use of beta weights only is not enough because they can change when more predictors are added or removed. Therefore, predictor variables cannot independently predict the value of the dependent variable if they are highly correlated, causing multicollinearity (Fox and Monette, 1992). Multicollinearity can be detected using tolerance and variance inflation factor (VIF). A small tolerance and a high VIF value denote high collinearity (Hair et al., 1998). A tolerance value under 0.20 suggests very high multicollinearity (Weisburd and Britt, 2014), and predictors with VIF values greater than 3.3 should be excluded (Diamantopoulos and Siguaw, 2006). Results indicated that multicollinearity was not a concern (highest education level: tolerance = 0.63, VIF = 1.60; age: tolerance = 0.83, VIF = 1.20; internet use frequency: tolerance = 0.68, VIF = 1.48).

## Discussion and Implication

Due to people’s busy daily schedules, increasing use of ICT is adopted to provide public services. In this context, Lessa (2019) showed that, in developing countries, there is low growth in terms of eGovernment initiatives. Similarly, recent studies have explored eGovernment challenges (Kumar et al., 2007; Glyptis et al., 2020; Almuraqab, 2021) but have not presented hypothetical clarifications of how and why this affects SWB. The present study’s objective is to investigate the influence of eGovernment efficiency on individuals’ SWB. We followed the theory of human covert and overt behavior depicted by the SOBC model (Davis and Luthans, 1980) to address our research objective. In the process, the study diversifies and adds explanations in terms of the relationship between eGovernment efficiency and SWB in a more detailed, fundamental, and precise way.

Prior research has investigated eGovernment efficiency’s impacts and has revealed it as the leading cause of challenges, failures, and successes. Based on this significant aspect, the present study explores the relationship between eGovernment efficiency and covert and overt behavior dimensions, revealing that eGovernment efficiency is significantly positively associated with covert behavior (trust). Due to online public service delivery benefits, users interact with the systems, thus experiencing how efficient they are. Therefore, if the service satisfies users’ expectations, users trust the system.

According to Kahneman (2011), trust can alter one’s behavior as a covert stimulus. It influences users to utilize eGovernment for services, mainly because it concerns service delivery standards, such as ideals and expectations. The level of service satisfaction triggers a specific influence on SWB. The greater the satisfaction level, the greater the positive effect on SWB. Positive SWB is associated with frequent satisfaction (LS), frequent positive affect (PA), and infrequent negative affect (NA) (Andrews, 1974). These feelings are essential to individuals’ overall health, empowering them to overcome complications positively and accomplish what they want from life (Diener and Chan, 2011).

### Theoretical Recommendation

This study makes several theoretical contributions. First, we implemented the SOBC model to construct a theoretical concept of the antecedents, mediators, and consequences of eGovernment efficiency concerning citizens’ SWB. Most previous studies have adopted the TAM and UTAUT frameworks to examine the relationship between eGovernment and citizens. However, the present study utilized the SOBC model, a comprehensive model of human behavior, to explore this multifaceted human behavior better. Second, according to the SOBC model, trust is a cognitive process that plays an essential mediating role in explaining the consequences of human behavior. The present study’s results support this concept of the SOBC model; trust mediates the relationship between eGovernment efficiency and utilization, which then triggers effects on SWB. Essentially, when eGovernment is efficient, and utilization is effective, this results in either two of the three SWB components [satisfaction (LS) and positive affect (PA)]; if not, this results in negative affect (NA) (Andrews, 1974; Diener and Emmons, 1984). Third, focusing on these different mediating effects of trust, we emphasize the moderating impact of socio-demographic characteristics in exploring variation in trust. The stepwise multiple regression results show that the highest education level, age, and internet access frequency together moderate trust. Information technology usage has expanded intensively; therefore, gender does not influence performance expectancy (Goswami and Dutta, 2015).

### Practical Recommendation

The findings from this study reveal practical suggestions for developing systems that consider users’ feelings and behavior patterns to encourage trusting behavior and ultimately have a more significant favorable influence on users’ SWB. Despite the known orotundity concept that the best way to prevent technology issues is not to use it as much (Whelan et al., 2020), this concept does not apply when vital public services are mandatory online only. Since governments provide constraints in citizens’ day-to-day behavior to shield them from foreign intrusion and regularly cater for their happiness and overall wellbeing (Traill et al., 2014), the same objectives should be considered in eGovernment service delivery. Future research should examine whether interventions intended to make eGovernment efficient incorporate factors such as privacy, security, citizen and social engagement, content, services, and usability (Holzer and Kim, 2006). These factors are essential to strengthen the relationship between the online government environment and trust development. In addition, future research can explore the effectiveness and efficiency of online banking systems that enhance service quality, customer satisfaction, customer trust, and loyalty (Yoon, 2010; Chu et al., 2012) and benchmark insights and merge them into eGovernment systems to enhance efficiency. By so doing, government agencies will be able to accommodate the positive wellbeing of citizens concerning accessing essential public services online. Since demographic characteristics, such as internet use frequency and highest education level, influence trust in eGovernment, exposure and education significantly impact the utilization rate. This study recommends implementing eGovernment service access and using training programs and channels specifically formulated for citizens to affect and increase digital literacy positively. Since more people use social media daily, this could be achieved by posting short videos tutorials on government agencies’ social media pages. Such initiatives could provide more opportunities to citizens and promote the unbiased usage of public services in developing communities.

### Limitations and Future Research

Just like any empirical research, this study has limitations. First, while this study implemented the SOBC theory to explain the measures and consequences of eGovernment efficiency, the variance explained by the study model is reasonable. Further investigations can examine, validate and argue whether other variables aligning with the SOBC model have more predictive power. For instance, different cognitive processes may mediate the relationship between eGovernment efficiency and SWB.

Second, the study used primary data with appropriate sample data corresponding with the social science statistical rule of thumb. However, this could limit the generalization of the findings because questionnaire responses depend on the respondents’ current mood (Siniscalco and Auriat, 2005). However, Lessa (2019) review indicates that previous research has documented eGovernment challenges in developing countries. This study, therefore, suggests that future research should validate the present research results using secondary data, e.g., reviews and other official records from government agencies and a different SEM methodology such as partial least squares (PLS).

Third, as we gathered data from users in one developing country (Botswana), cultural differences may affect the study’s findings (Polkinghorne, 2005) since different cultures have different habits, online experiences, education structures, and internet frequency. Network infrastructures challenges elevate doubt or lack of trust in utilizing e-services (Samboma, 2019). During data collection, when contacted, some respondents said, “*I will fill the survey as soon as I have internet access*.” Due to this, we noticed that internet services hindered our survey accessibility which is mainly the same challenge toward accessing other e-services. Ntshwarang et al. (2021) pointed to infrastructure and poor internet access off-campus as the main challenges faced concerning electronic learning in the University of Botswana during the COVID-19 pandemic. Therefore, future research should consider this direction, possibly by comparing two or more developing countries, or even including developed countries, to validate the present study’s results using a bigger sample size.

## Data Availability Statement

The raw data supporting the conclusions of this article will be made available by the authors, without undue reservation.

## Author Contributions

MF and ME: conceptualization. SAQ, ME, and NAQ: methodology. ME and SAQ: software. MF, SAQ, ME, and NAQ: validation. ME and SAQ: formal analysis. ME and SAQ: investigation. MF: resources. ME: data curation. MF and SAQ: writing (original draft preparation). SAQ: writing (review and editing). NAQ and ME: visualization. MF: supervision. MF, ME, and SAQ: project administration.

## Conflict of Interest

The authors declare that the research was conducted in the absence of any commercial or financial relationships that could be construed as a potential conflict of interest.

## Publisher’s Note

All claims expressed in this article are solely those of the authors and do not necessarily represent those of their affiliated organizations, or those of the publisher, the editors and the reviewers. Any product that may be evaluated in this article, or claim that may be made by its manufacturer, is not guaranteed or endorsed by the publisher.
