# Supplementary material for: The Effects of eGovernment Efficiency on Subjective Wellbeing
Source: Front Psychol. 2022 Mar 3;13:768540. doi: 10.3389/fpsyg.2022.768540 (PMC8928557; doi:10.3389/fpsyg.2022.768540)
Supplement: Supplementary file 1 [file Data_Sheet_1.docx]

## **Appendix 1. Demographics of the respondents**

| **Sample Characteristics** |  | **Percentage** |
| --- | --- | --- |
| Sector | Private sector | 26 |
|  | Government sector | 18 |
|  | University student | 31 |
|  | Unemployed | 25 |
| Internet use frequency | Once a month | 2 |
|  | Fortnightly | 1 |
|  | Twice a week | 19 |
|  | Daily | 78 |
| Highest education level | Never attended school | 0 |
|  | Primary/elementary school | 2 |
|  | Junior school | 6 |
|  | High school | 19 |
|  | Tertiary education | 73 |
| Gender | Male | 57 |
|  | Female | 43 |
| Age (years) | 18–24 | 3 |
|  | 25–34 | 46 |
|  | 35–44 | 33 |
|  | 45–54 | 17 |
|  | 55–64 | 1 |

**Note:** *N* = 300.

## **Appendix 2. Descriptive statistics of scale variables**

| **Variable** | **Min.** | **Max.** | **Range** | **Mean** | **SD** | **Skewness** | **Kurtosis** |
| --- | --- | --- | --- | --- | --- | --- | --- |
| Q1 | 2 | 5 | 3 | 3.677 | 0.606 | –0.523 | 0.328 |
| Q2 | 2 | 5 | 3 | 3.737 | 0.549 | –1.390 | 1.924 |
| Q3 | 2 | 5 | 3 | 4.000 | 0.597 | –0.800 | 1.837 |
| Q4 | 2 | 5 | 3 | 3.823 | 0.541 | –0.486 | 0.946 |
| Q6 | 2 | 5 | 3 | 3.703 | 0.764 | –0.351 | –0.351 |
| Q7 | 2 | 5 | 3 | 3.850 | 0.670 | –0.488 | 0.636 |
| Q8 | 2 | 5 | 3 | 3.867 | 0.608 | –0.554 | 1.163 |
| Q9 | 1 | 5 | 4 | 3.857 | 0.619 | –0.661 | 1.872 |
| Q10 | 2 | 5 | 3 | 3.820 | 0.624 | –0.603 | 1.026 |
| Q11 | 1 | 5 | 4 | 3.996 | 0.636 | –0.109 | 3.468 |
| Q12 | 2 | 5 | 3 | 4.070 | 0.683 | –0.596 | 0.866 |
| Q13 | 2 | 5 | 3 | 4.097 | 0.675 | –0.381 | 0.140 |
| Q14 | 1 | 5 | 4 | 3.963 | 0.671 | –1.094 | 3.226 |
| Q15 | 1 | 5 | 4 | 4.317 | 0.762 | –0.994 | 1.731 |
| Q16 | 1 | 5 | 4 | 4.317 | 0.724 | –1.201 | 2.289 |
| Q17 | 2 | 5 | 3 | 4.363 | 0.673 | –0.917 | 1.015 |
| Q18 | 2 | 4 | 2 | 3.530 | 0.625 | –0.984 | –0.082 |

**Note:** *N* = 300.

## **Appendix 3. Parameter estimates from the structural equation model**

| **Explained Variable** | **Predictor Variable** | **Estimate** | **Std. err.** | **Value** | ***p*-Value** |
| --- | --- | --- | --- | --- | --- |
| Trust | eGovernment efficiency | 0.828 | 0.148 | 5.573 | *** |
| Utilization | Trust | 0.483 | 0.063 | 7.701 | *** |
| SWB | Utilization | 0.914 | 0.101 | 9.034 | *** |
| eGovernment efficiency | Q1 | 1.000 |  |  |  |
|  | Q2 | 0.874 | 0.136 | 6.419 | *** |
|  | Q3 | 0.987 | 0.151 | 6.525 | *** |
|  | Q4 | 0.710 | 0.124 | 5.723 | *** |
| Trust | Q6 | 1.000 |  |  |  |
|  | Q7 | 0.950 | 0.072 | 13.193 | *** |
|  | Q8 | 0.822 | 0.065 | 12.642 | *** |
|  | Q9 | 0.791 | 0.066 | 11.950 | *** |
| Utilization | Q10 | 1.000 |  |  |  |
|  | Q11 | 0.869 | 0.106 | 8.211 | *** |
|  | Q12 | 0.974 | 0.112 | 8.727 | *** |
|  | Q13 | 0.922 | 0.119 | 5.723 | *** |
| SWB | Q14 | 1.000 |  |  |  |
|  | Q15 | 1.099 | 0.095 | 11.557 | *** |
|  | Q16 | 0.981 | 0.090 | 10.907 | *** |
|  | Q17 | 0.767 | 0.083 | 9.198 | *** |
|  | Q18 | 0.379 | 0.780 | 4.840 | *** |

**Note:** *** *p* ≤ 0.001.
